# Methodology: an optimized, high-yield tomato leaf chloroplast isolation and stroma extraction protocol for proteomics analyses and identification of chloroplast co-localizing proteins

**DOI:** 10.1186/s13007-020-00667-5

**Published:** 2020-09-24

**Authors:** Oindrila Bhattacharya, Irma Ortiz, Linda L. Walling

**Affiliations:** Department of Botany and Plant Sciences, Center for Plant Cell Biology, University of California, Riverside, CA 92521 USA

**Keywords:** *Solanum lycopersicum*, Chloroplast isolation, Stroma, Soluble proteins, Proteomics

## Abstract

**Background:**

Chloroplasts are critical organelles that perceive and convey metabolic and stress signals to different cellular components, while remaining the seat of photosynthesis and a metabolic factory. The proteomes of intact leaves, chloroplasts, and suborganellar fractions of plastids have been evaluated in the model plant Arabidopsis, however fewer studies have characterized the proteomes of plastids in crops. Tomato (*Solanum lycopersicum*) is an important world-wide crop and a model system for the study of wounding, herbivory and fruit ripening. While significant advances have been made in understanding proteome and metabolome changes in fruit ripening, far less is known about the tomato chloroplast proteome or its subcompartments.

**Results:**

With the long-term goal of understanding chloroplast proteome dynamics in response to stress, we describe a high-yielding method to isolate intact tomato chloroplasts and stromal proteins for proteomic studies. The parameters that limit tomato chloroplast yields were identified and revised to increase yields. Compared to published data, our optimized method increased chloroplast yields by 6.7- and 4.3-fold relative to published spinach and Arabidopsis leaf protocols, respectively; furthermore, tomato stromal protein yields were up to 79-fold higher than Arabidopsis stromal proteins yields. We provide immunoblot evidence for the purity of the stromal proteome isolated using our enhanced methods. In addition, we leverage our nanoliquid chromatography tandem mass spectrometry (nanoLC–MS/MS) data to assess the quality of our stromal proteome. Using strict criteria, proteins detected by 1 peptide spectral match, by one peptide, or were sporadically detected were designated as low-level contaminating proteins. A set of 254 proteins that reproducibly co-isolated with the tomato chloroplast stroma were identified. The subcellular localization, frequency of detection, normalized spectral abundance, and functions of the co-isolating proteins are discussed.

**Conclusions:**

Our optimized method for chloroplast isolation increased the yields of tomato chloroplasts eightfold enabling the proteomics analysis of the chloroplast stromal proteome. The set of 254 proteins that co-isolate with the chloroplast stroma provides opportunities for developing a better understanding of the extensive and dynamic interactions of chloroplasts with other organelles. These co-isolating proteins also have the potential for expanding our knowledge of proteins that are co-localized in multiple subcellular organelles.

## Background

Plastids control key metabolic processes central to cell vitality and function. Plastid forms, chemistry and molecular operations are dynamic and are influenced by developmental and environmental cues [1–5]. In young and mature green leaves, chloroplasts predominate and serve as the sites of many critical biological functions such as photosynthesis, carbon fixation, nitrogen and sulfur assimilation, chlorophyll biosynthesis and breakdown, and synthesis of a wide range of biomolecules (e.g. amino acids, fatty acids, lipids, tocopherols, carotenoids, purine and pyrimidine nucleotides, tetrapyrroles, and isoprenoids) [2, 6]. Furthermore, numerous plant hormones (e.g., jasmonic acid, salicylic acid, gibberellic acid, abscisic acid, cytokinin, and brassinosteroids) with key roles in defense and development initiate their biosynthesis in this organelle [3, 6].

The multi-copy, stroma-localized chloroplast genome encodes proteins that have critical roles in the control of chloroplast gene expression (e.g., transcriptional and translational machinery) and assembly of the multimeric complexes for photosystem I and II, as well as other functions [3, 7–9]. Surprisingly, of the 80–100 proteins encoded by the plant chloroplast genomes, relatively few directly contribute to the diverse metabolic paths active within this organelle. A majority of the proteins that control chloroplast gene expression, photosynthesis, protein turnover, and the chloroplast’s biochemical diversity are derived from nuclear genome-encoded proteins that are imported into the chloroplast [3, 10–12]. These imported proteins reside within one of the chloroplast’s membrane systems (e.g., the inner and outer membranes of the envelope or the thylakoid membranes) or localize into one of its compartments (e.g., the stroma or lumen). The chloroplast envelope not only forms a barrier between the cytosol and the stroma, it has integral membrane transporters to mediate water, ion and metabolite transport to and from the stroma [13]. Furthermore, the chloroplast envelope harbors the protein transport machinery (e.g., the TIC and TOC complexes) that facilitates the import of thousands of proteins into the chloroplast [11, 12, 14]. This canonical path for protein import into the chloroplast predominates and relies on N-terminal transit peptides to expedite precursor protein import into the chloroplast. However, there are non-canonical paths for protein entry into chloroplasts and other plastid forms [1, 15]. The massive influx of nuclear genome-encoded proteins is consistent with the cyanobacterial origins of chloroplasts, and the evolutionary trend for plastid genome reduction and relocation of genes from the plastid genome to the nuclear genome for expression [16, 17].

Chloroplasts also play a central role in organellar communication, as regulatory hubs capable of sensing and relaying changes in organellar and cellular homeostasis. Chloroplasts communicate with the nucleus via biogenic and operational signals to modulate nuclear gene expression during chloroplast biogenesis and in response to stress, respectively. This chloroplast-to-nucleus communication (retrograde signaling) tightly coordinates cellular processes with chloroplast activities [5, 18–20]. Retrograde signals have been primarily studied in the model plant *Arabidopsis thaliana*. A wide variety of metabolites including adenosine derivatives [21], reactive oxygen species [22, 23], chlorophyll precursors [24–26], an isoprenoid precursor [27], oxidation products of beta-carotene [28], and transcription factors [29, 30] have been shown to directly mediate retrograde signaling during stress. Chloroplasts also have an intimate interaction with peroxisomes, mitochondria and the endoplasmic reticulum allowing metabolite exchange and rapid signaling between these cellular compartments to orchestrate responses to cellular stress [31, 32].

We are at the threshold to understanding the plethora of retrograde signals being generated by the chloroplast, as this research space is in its infancy in model plants, as well as in crop plants. In tomato, a stroma-localized enzyme called leucine aminopeptidase A (LAP-A) controls retrograde signaling after herbivory, wounding and methyl-jasmonate treatment [33]. LAP-A is critical for the activation of wound-response genes (e.g., proteinase inhibitors and polyphenol oxidase) and is a repressor for several pathogenesis-related protein genes and chaperones [33, 34]. LAP-A is bifunctional—it is an aminopeptidase and a molecular chaperone [35–38]. By trimming N-terminal amino acids from stroma-localized proteins or peptides and/or maintaining the native folding status of stromal proteins, LAP-A acts post-translationally to generate or maintain a retrograde signal. The LAP-A-dependent retrograde signal is likely controlled by one or more of the estimated 3000 proteins imported into chloroplasts or chloroplast genome-encoded proteins (the chloroplast proteome) [7]. Therefore, our laboratory is taking a multi-pronged proteomics approach to identifying LAP-A substrates. Two of these strategies focus on the analysis of the protein complement in the stroma of wild-type, *LapA*-silenced and *LapA*-overexpressing plants and the changes in the stromal N-terminome by nanoLC-MS/MS. These experiments in conjunction with the identification of LAP-A-binding proteins should reveal the identity of the LAP-A-regulated retrograde signal.

Prior to embarking on the three proteomic-based strategies, high-yielding methods for isolation of tomato chloroplasts and stroma were needed. Methods for isolating chloroplasts of proteomics quality from Arabidopsis, pea, spinach, and several monocots have been described [39–46]. In addition, methods for tomato chromoplast isolation have been described and utilized in describing chromoplast transitions during fruit development [47–51]. In contrast, the methods for proteomics-grade tomato chloroplasts are limited [52]. Here we describe a protocol for isolating tomato chloroplasts and stromal proteins for proteomics analyses. We empirically tested several parameters, which afforded marked yield increases in tomato chloroplasts and stroma for proteomics analyses. We assess the quality of our tomato chloroplast stromal proteome by immunoblot analyses to monitor the presence of proteins from different chloroplast subcellular compartments. We also provide the proteomics decision pipeline used for identifying stromal proteins and 254 proteins that reproducibly co-isolate with the tomato chloroplast stroma.

## Materials and methods

Note: This protocol is scaled for three 60-g leaf chloroplast preparations.

### Reagents for chloroplast and stroma isolation

- Bovine serum albumin (BSA) (Sigma-Aldrich, catalog #A7030)
- D-sorbitol (Sigma-Aldrich, catalog #S1876)
- EDTA (Ethylenediaminetetraacetic acid; Sigma-Aldrich, catalog #E9884)
- Ficoll-400 (Sigma-Aldrich, catalog #F2637)
- HEPES (4-(2-hydroxyethyl)-1-piperazineethanesulfonic acid; Sigma-Aldrich, catalog #H3375)
- MgCl_2_.6H_2_O (Sigma-Aldrich, catalog #M9272)
- MnCl_2_ (Sigma-Aldrich, catalog #244589)
- Percoll (GE Healthcare Life Sciences, catalog #17-0891-01)
- Poly(ethyleneglycol)-8000 (PEG-8000; Sigma-Aldrich, catalog #1546605)
- Potassium hydroxide (Sigma-Aldrich, catalog #221473)
- Proteinase inhibitor cocktail for plant cell and tissues extracts (Sigma-Aldrich, catalog #P9599). Store at − 20 °C until use.
- Sodium ascorbate (Sigma-Aldrich, catalog #A7631)

### Glassware, plasticware, consumables, and equipment for isolation of chloroplasts

Note: All glassware, plasticware, consumables, and equipment should be prechilled to 4 °C.

- Graduated glass cylinders (one 1-L, one 500-mL, two 100-mL, one 50-mL, sterile)
- Conical flasks (three 1-L, sterile)
- Wide-mouthed glass funnels (three, sterile)
- Beakers (one 1-L, one 2-L, four 500-mL, two 100-mL, one 50-mL, sterile)
- Glass bottles (one 500-mL, one 200-mL, one 50-mL, sterile)
- Disposable screw-cap tubes (three 50-mL, three 15-mL, sterile)
- Nitex squares (six 12" x 12" squares, 60-μm or 90-μm mesh)
- Glass plates (two, for chopping leaves)
- Test tube racks for 30-mL glass tubes (two, one must fit in ice bucket)
- Glass centrifuge tubes (Corning) (twelve 30-mL, sterile)
- Centrifuge bottles for a Beckman JS 5.3 rotor (four 250-mL, sterile)
- High-speed swinging bucket centrifuge, rotors and adaptors (Prechill at 4 °C)
-
  - Beckman-Coulter Avanti J-26 XP Centrifuge (Beckman-Coulter, catalog #393124)
  - JS 5.3 rotor (Beckman-Coulter, catalog #368690)
  - Adapters for 30-mL tubes (two, Beckman-Coulter, catalog #392076)
  - Rubber sleeves for 30-mL Corning centrifuge tubes (six, Corning, catalog #8441)
  - Adapters for 250 mL-centrifuge bottles (four, Beckman-Coulter, catalog #392077).
- Aluminum foil
- Nitrocellulose filters (0.45-μm; Thermo Fisher Scientific, catalog #121-0045)
- Blender and 250-mL cup (Waring, catalog #7012S and MC3, respectively)
- Microcentrifuge tubes (three 2-mL, Axygen, catalog #MCT-200-C, sterile)
- Ice buckets
- Metal spatulas
- Paint brushes (Ultra soft-tipped, #10)
- Parafilm
- Pasteur pipettes (six, sterile)
- Pipetmen (P1000, P200, P20) and sterile tips
- Pipettes (plastic, sterile; 5-mL and 10-mL)
- Pipette pump for 10-mL pipettes (Bel-Art Products)
- Razor blades
- 37 °C water bath
− 20 °C freezer
− 80 °C freezer
- Markers for labeling tubes and bottles

### Glassware, plasticware, consumables, and equipment for isolation of stroma

- Beakers (one 100-mL)
- Amicon Ultra-2 Centrifugal Filter Unit with Ultracel-3 membrane (EMD Millipore catalog #UFC200324) (four, with one as a balance)
- Microcentrifuge tubes (three 2-mL, Axygen, catalog # MCT-200-C, sterile)
- Beckman-Coulter Avanti J-26 XP Centrifuge
-
  - JS 5.3 rotor; two adapters for 30-mL tubes (Beckman-Coulter, catalog #392076)
  - Rubber sleeves for 15-mL Corning centrifuge tubes (four, prechill at 4 °C)
- Beckman-Coulter Optima™ MAX-TL Ultracentrifuge (Beckman-Coulter, Catalog #A95761)
-
  - TLA-100.3 fixed angle ultracentrifuge rotor (Beckman-Coulter, Catalog #349481) (Prechill 4 °C)
  - Polycarbonate ultracentrifuge tubes, 3.5-mL, thick-walled (Beckman-Coulter, Catalog #349622) (three, prechill at 4 °C)
- Microfuge (4 °C)
- Microfuge tubes, 1.5-mL (12, sterile)
- Disposable 15-mL tube (one, sterile)
- Tissue grinder (Tenbroeck 2-mL capacity, Corning Life Sciences, part #7727-2)
- Pipetmen (P1000, P200, and P20) and tips
- Wide-bore 1-mL pipette tips: Using a razor blade, cut 2 mm off the tip of 1-mL pipetman tips. A minimum of three wide-bore tips will be needed per chloroplast preparation. Wide-bore tips are stored in a 1-mL pipette-tip box and autoclaved.
- Vortexer
- Ice bucket
- Marking pens for labeling and marking tubes

### Stock solutions for chloroplast isolation (1 week in advance)

Note: For stock solutions for proteomics preparations, gloves are worn continuously to avoid common protein contaminants.

1 M HEPES–KOH (pH 8). Make 100 mL. Filter through a 0.45 µM filter into a sterile bottle.

0.5 M EDTA-NaOH (pH 8). Make 100 mL. Adjust pH with NaOH. Autoclave.

1 M MgCl_2_. Make 100 mL. Autoclave.

1 M MnCl_2_. Make 100 mL. Autoclave.

Percoll-PEG-Ficoll (PPF) stock: Autoclave 100 mL of Percoll in a 200-mL glass bottle and cool. To compensate for water loss after autoclaving, adjust Percoll volume to 100 mL with sterile deionized water. The stock can be stored until the day of use. Three to four hr prior to use, add PEG-8000 to 3% (w/v) and Ficoll-400 to 1% (w/v) to the volume-adjusted Percoll. Mix well and store at 4 °C until use.

10X Percoll Gradient Buffer (PGB): Prepare 20 mL of 10 X PGB (500 mM HEPES–KOH (pH 8), 3.3 M D-sorbitol, 20 mM EDTA (pH 8), 10 mM MgCl_2_, 10 mM MnCl_2_) in a 50-ml sterile bottle. Use the 1 M HEPES–KOH (pH 8), 1 M EDTA, 1 M MgCl_2_, and 1 M MnCl_2_ stocks, and D-sorbitol. Place in a 37 °C water bath to dissolve sorbitol. Add BSA and sodium ascorbate to a final concentration of 2.5% (w/v) BSA and 1% (w/v) sodium ascorbate. If needed, adjust volume with sterile water to 20 mL. Store at 4 °C until use.

Sterile deionized water: Three to four L for preparing working buffers and soaking Nitex squares. Store at 4 °C until use.

### Working solutions for chloroplast preparation (day of chloroplast isolation)

1X Grinding Buffer (1X GB) (Make 4 h prior to leaf homogenization): In a sterile 2-L beaker, make two liters of 1X GB (50 mM HEPES–KOH (pH 8), 330 mM D-sorbitol, 2 mM EDTA-NaOH (pH 8), 1 mM MgCl_2_, 1 mM MnCl_2_, 0.25% BSA (w/v), and 0.1% (w/v) sodium ascorbate) using stock solutions of 1 M HEPES–KOH (pH 8.0), 0.5 M EDTA-NaOH (pH 8), 1 M MgCl_2_, and 1 M MnCl_2_. Add D-sorbitol and mix thoroughly. Add BSA and sodium ascorbate. Transfer 10 mL of 1X GB to a 50-mL beaker, cover with an aluminum foil cap and store at 4 °C. Cover the 2-L beaker with aluminum foil and transfer beaker to -20 °C for 4 h to produce an ice slush.

The 1:1 ice:liquid ratio is one of the key factors for recovering high yields of tomato chloroplasts. The 1X GB at -20 °C must be vigorously stirred with a sterile pipette periodically (immediately prior to leaf harvest, midway during leaf harvest and prior to tissue grinding) to obtain equal parts ice and liquid. After stirring, return 1X GB to -20 °C freezer.

1X HEPES-Sorbitol buffer (1X HS) (3–4 h prior to leaf homogenization): Make 500 mL of 1X HS (50 mM HEPES–KOH (pH 8), 330 mM D-sorbitol). Use the 0.5 M HEPES–KOH (pH 8.0) stock and add D- sorbitol to sterile deionized water to a volume of 500 mL in a sterile 500-mL bottle. Mix well and store at 4 °C until use.

Percoll step gradients (30 min prior to leaf homogenization): Using the PPF stock, 10X PGB, and sterile deionized water, prepare 64 mL of 40% Percoll in 1X PGB and 34 mL of 80% Percoll in 1X PGB in 100-mL beakers. Assemble six 40%-80% Percoll step gradients (two gradients per 60-g leaf chloroplast preparation). Dispense five mL of the 80% Percoll-1X PGB (80% Percoll cushion) at the bottom of six prechilled, 30-mL glass centrifuge tubes. Using a sterile 10-mL pipette and a 10-mL pipette pump, draw up 10 mL of the 40% Percoll-1X PGB into the pipette slowly; avoid air bubbles. Hold a 30-mL tube with its 80% Percoll cushion at a 45^o^ angle; rest the tube base on the bench top if needed. Insert the pipette tip into the tube. The pipette is angled so the body of the pipette contacts the upper lip of the tube. Gently break the seal between the pipette pump and the pipette; this allows the 40% Percoll solution to gently flow down the side of the tube in a thin stream and layer over the 80% Percoll cushion forming a sharp well-defined interface. The pipette is gradually lifted as the 40% Percoll solution is delivered. It is critical that there is no mixing at the interface; an undisturbed 40–80% Percoll interface increases yields of intact chloroplasts, which are located at this interface after centrifugation. Cover the Percoll gradients with aluminum foil, place in test tube rack embedded in an ice bucket with ice slush and store in the cold room until use.

Nitex squares (1–2 h prior to leaf homogenization): Soak six Nitex filters in a 1-L beaker of sterile water prior to use and store at 4 °C.

### Stock solutions for stroma isolation (1 week in advance)

500 mM HEPES–KOH (pH 8): Make 100 mL adjust pH with KOH pellets. Sterile filter into sterile 100-mL storage bottle.

250 mM MgCl_2_: Make 100 mL and autoclave.

Sterile deionized water: < 100 mL for preparing working buffers and preparing Amicon filtration systems.

### Plant growth

For one large-scale chloroplast preparation (~ 60 g), leaves from 18 five-week-old plants are harvested. Tomato seeds (*Solanum lycopersicum* UC82b) are surface sterilized in 10% (v/v) bleach for 5 min. Following three 5-min washes in sterile water, seeds are placed on water-moistened Whatman #1 filter paper disc in petri dishes. Seeds are allowed to germinate for seven days at room temperature with 75 µmol m^−2^ s^−1^ light. Seedlings are transferred to UC Soil Mix 3 in flats with 18-section inserts (McConkey Growers Catalog # EJP1801-200) and grown in a growth chamber for four weeks at 28 °C for 16 h (day) and 22 °C for 8 h (night). Lights were maintained between 350 and 450 μmol m^−2^ s^−1^. Plants are watered daily and fertilized weekly with a 0.35% (w/v) MiracleGro Tomato Plant Food solution. Plants with three to four true leaves are used for chloroplast isolation. Twenty-seven hours prior to the chloroplast isolation, tomato plants are transferred to the dark to reduce starch. Starch-filled chloroplasts lyse and reduce yields of intact chloroplasts.

### Chloroplast Isolation Protocol

#### General Comments

All stock solutions, buffers, equipment, glassware, and plastic ware are chilled (4 °C) prior to use. Solutions are autoclaved or sterile filtered. If chloroplasts are to be used for proteomics studies, all steps should be performed wearing latex gloves. All steps of the protocol are carried out quickly in the cold room. An ice slush is used for chilling and transporting centrifuge bottles and tubes within ice buckets. This protocol is scaled for performing three 60-g leaf chloroplast preparations simultaneously.

**Fig. 1:**
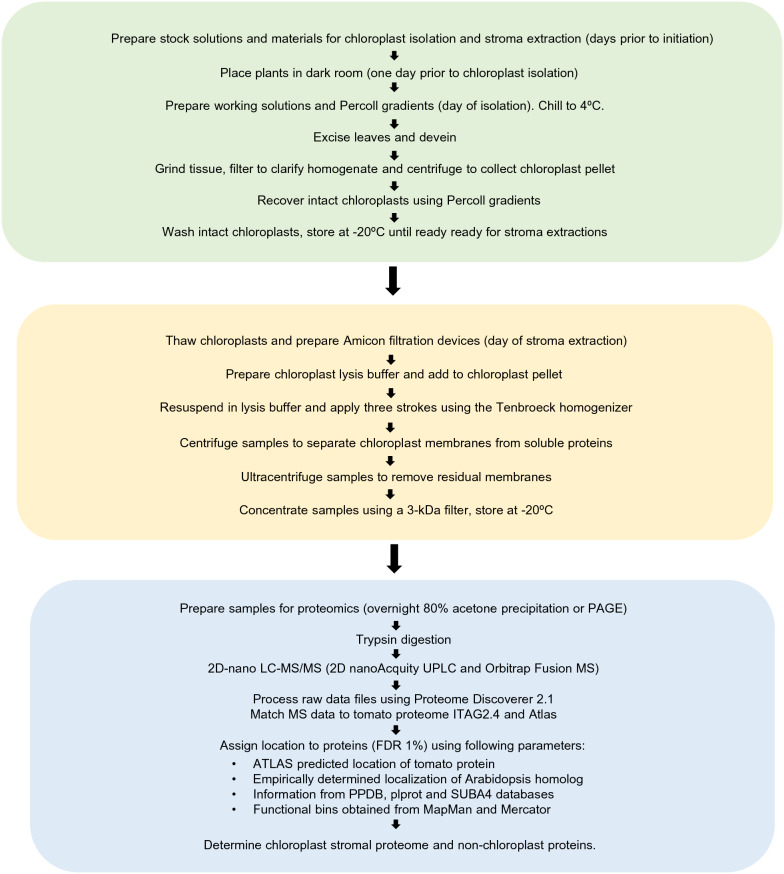
Flowchart of events for chloroplast isolation, stroma extractions and proteomics data generation and analyses. The major steps for chloroplast isolation (green), stroma extractions (yellow) and proteomics analyses (blue) are outlined and described in detail in “Materials and methods”

To develop our chloroplast isolation protocols, we considered the published protocols for isolating tomato leaf chloroplasts for proteomics [52], protein import and biochemistry [53, 54], and chloroplast genome isolation [55–57], as well as methods for plastid isolation from tomato fruit [48–50, 58] (Additional file 1: Table S1). While protoplasts clearly provide the highest chloroplast yields [59], the lengthy preparation times and use of cell wall hydrolyzing enzymes are disadvantageous to proteomics studies studying biotic stress, as this method generates cell wall-derived elicitors that could trigger plant-defense responses (Table 1). Our protocol primarily builds upon the Arabidopsis chloroplast isolation methods and stromal protein isolation methods of van Wijk et al. [42] with several modifications as discussed here or in the “Comments” section. Figure 1 provides a flow chart that summarizes the basic sequence of events for: (1) the isolation of tomato chloroplasts, (2) isolation of chloroplast stromal proteins, and (3) proteomics sample processing and data analysis. In addition to the detailed protocols below, we provide a streamlined workflow checklist for chloroplast isolation that can easily be used for tracking steps (Additional file 2: Table S2).

**Table 1:** Chloroplast and stromal protein yields

Preparation	Chloroplast Yields (µg Chl/g FW)	Stromal Protein Yields (µg stromal protein/g FW)	Fold change (relative to tomato leaf #1)	References
Tomato leaf #1^a^	5.61	1.56	1	This paper
Tomato leaf #2^b^	19.15	5.3	3.4	This paper
Tomato leaf #3^c^	26.50	7.95	4.7	This paper
Tomato green fruit plastids	2.7	–	0.5	Suzuki et al. [51]
Arabidopsis protoplasts	50–100	–	8.9–17.8	Fitzpatrick and Keegstra [59]
Arabidopsis leaves	5	–	1.1	Fitzpatrick and Keegstra [59]
Arabidopsis leaves	–	0.1–0.2		Hall et al. [46]
Spinach leaves	4–5	–	0.7–1.1	Rensick et al. [60]

^a^The tomato leaf #1 chloroplast prep was performed prior to optimization of blender time and speed and ice slurry consistency. Leaf segments were added in two batches into the blender

^b^The tomato leaf #2 chloroplast prep was performed with optimized blender time and speeds. Leaf segments were added in two batches into the blender

^c^The tomato leaf #3 chloroplast prep was performed with optimization of blender time and speed and ice slurry consistency. Leaf segments were added in two batches into the blender

Prior to initiating this protocol, it is critical to empirically determine the optimal blender setting and duration of blending that will be used in “Tissue grinding” section. The 1X Grinding Buffer:blender cup volumes and the brand and settings of the blender used are critical parameters. The configuration of the blades and rotations per minute of the blades for each blender will be different. The blender settings are increased incrementally until the blades rapidly mix the 1X Grinding Buffer (ice slush) and leaves within the blender cup. If the setting is too low, inadequate homogenization will occur (e.g., supernatant is clear). If the setting is too high, chloroplasts will be sheared, the homogenate will be dark green and foamy and yields will be significantly reduced. In an optimal homogenization protocol, the homogenate is pale green and a substantial amount of leaf debris remains and is captured by the Nitex filter. While longer homogenization times decrease the amount of leaf debris, chloroplast yields decline due to lysis of intact chloroplasts and the percentage of intact chloroplasts was typically 14.6% (Table 1; leaf prep #1). Using short, optimized blending times, intact chloroplast yields increased 3.4-fold from 5.6 µg to 19.2 µg Chl/g FW and the mean  % of intact chloroplasts increased 4.6-fold to 66.8% (Table 1; leaf prep #1 vs #2).

Additional parameters critical for high yields include: leaf age and texture, the tissue:buffer ratio, the amount of the ice to liquid ratio of the 1X Grinding Buffer. Similar to the Arabidopsis protocol [42] and unlike other tomato plastid preparations (Additional file 1: Table S1), we used a 1:10 tissue to buffer ratio to assure adequate buffering capacity and movement of tissue in the homogenization process. We also discovered that adding leaves in two sequential 7.5-g additions to 150 mL of buffer provided optimal homogenization. The high yields of this protocol are also dependent on the 1X Grinding Buffer having a 1:1 ice:liquid ratio (see “Working solutions*”* section). The optimization of these steps increased yields to 4.7-fold from our initial protocol (Table 1; leaf prep #1 vs #3).

#### *Leaf excision and processing* (2 h prior to leaf homogenization)

Transfer dark-treated tomato plants into a room without direct light. Excise young leaves with a razor blade; thick and dark-green older leaves and damaged leaves (of any age) are not used. Using a razor blade and a glass plate as a cutting surface, remove the midrib from each leaflet; this step is critical for efficient homogenization. Quickly chop leaves into 2 × 2-cm pieces and transfer to aluminum foil sheets resting on ice in an ice bucket. To minimize tearing of leaves, change razor blades frequently to assure a clean cut. After collection of approximately 15 gm of leaves (~ 4–5 plants), wrap the aluminum foil around the leaves and store the packet at 4 °C until processing of all plants is complete. It takes two people approximately 2 h to process three 18-plant sets (~ 60 g leaves/set).

#### *Tissue grinding* (Time estimate ~ 45 min)

All steps are performed as quickly as possible in the cold room. Fit three wide-mouthed funnels with two pieces of Nitex (pre-wetted with water) and place funnel in each 1-L flask.

Homogenize leaves in small batches to assure the optimal amount of shear to release (but not damage) chloroplasts. A 1:10 (w/v) tissue to 1 X GB buffer slush ratio is used. Add approximately 7.5 g of chopped, deveined leaves and 100 mL of 1X GB ice slush to a prechilled, 250-mL stainless-steel blender cup. Cover the blender cup with an aluminum foil “cap” (for ease of handling) and homogenize for 2 s. Add the remaining 7.5 g of chopped, deveined leaves and 50 mL of 1X GB ice slush to the homogenate, stir with a metal spatula and homogenize for an additional 2 s. If tissue is limiting, the brei can be returned to the blender cup and homogenized with 50 mL 1 X GB ice slush for 2 s to increase yields.

Pour the homogenate through two layers of Nitex and collect in the 1-L flask. Repeat the homogenization procedure with additional 7.5-g samples until all leaf material (~ 60 g) in a preparation is processed. Homogenates for each treatment are pooled and allowed to passively filter through Nitex. Meanwhile, rinse the blender cup with water and dry. Process the additional 60-g leaf preparations immediately.

Gently squeeze the Nitex to recover residual buffer from the leaf debris. Approximately 250–300 mL of homogenate will be recovered per 60-g leaf preparation. Remove 20 mL of the homogenate to a 50-mL disposable tube and store at -20 °C for chlorophyll and protein analyses. Pour approximately 200 mL of each homogenate into a prechilled, 250-mL centrifuge bottle (1 bottle per 60-g preparation). Balance bottles with 1 X GB as needed; use the extra centrifuge bottle as a balance. Centrifuge bottles at 3800 g for 4 min at 4 °C in a JS 5.3 rotor in a Beckman-Coulter Avanti J-26 XP centrifuge. Immediately pour off each light-green supernatant into its own 500-mL beaker and mark location of pellet. Add the remaining homogenate to its corresponding crude chloroplast pellet. Balance bottles and centrifuge as described above. Pour the supernatant for each 60-g preparation into its corresponding 500-mL beaker. The green pellets contain both intact and broken chloroplasts and other cellular components. Faint white starch rings will be observed at the bottom of the bottle. In early stages of protocol optimization, it is useful to quantify chlorophyll in the supernatant (see “General comments*”* section). To this end, remove 10 mL of the supernatant to a 15-ml disposable tube and store at -20 °C for chlorophyll quantification.

#### *Chloroplast recovery* (Time estimate - 1 h)

Add cold (4 °C) 1X GB (1.5 to 2 mL) to the chloroplast pellets. Use a paintbrush that is pre-wetted in cold 1X GB to gently resuspend the pellet until no clumps remain. At this point, the crude chloroplast suspension should be thin enough to drip off the tip of the paintbrush. Add an additional 0.5–1 mL of 1X GB and mix well. This dilutes the suspension, prevents chloroplast aggregation and facilitates sample loading onto the Percoll gradients, as well as assuring better separation of intact and broken chloroplasts.

**Fig. 2:**
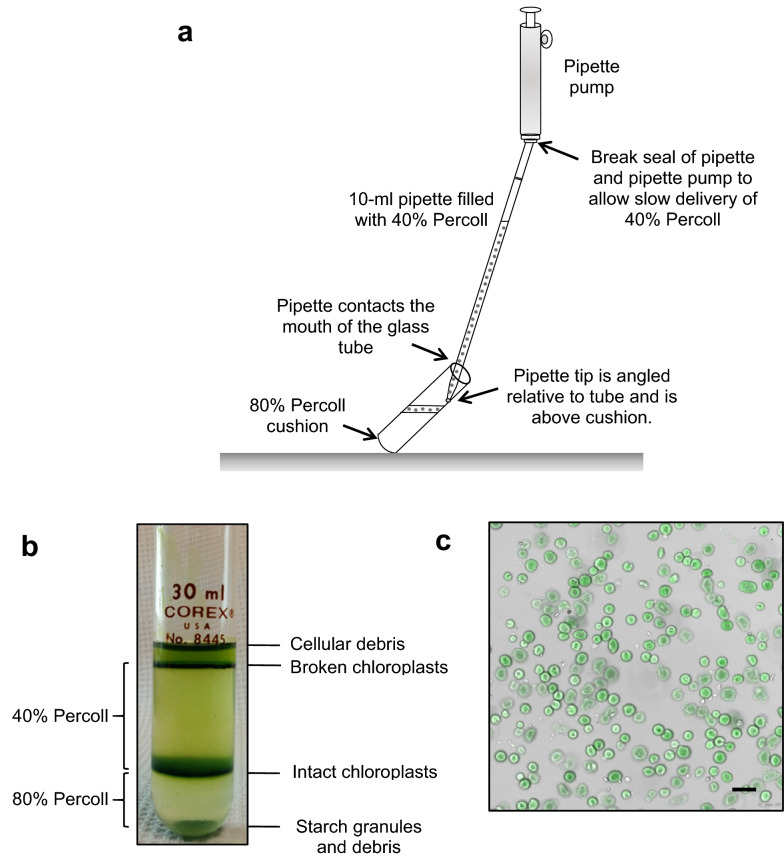
Isolation of tomato chloroplasts on Percoll gradients. **a** Minimal disturbance of the 40–80% interface is critical for high yields of intact chloroplasts. To assemble the Percoll gradient, a pipette containing 40% Percoll is angled relative to the tube and placed immediately above the 80% Percoll cushion. The seal of the pipette and pipettor is slowly broken allowing gentle layering of the 40% Percoll solution over the 80% Percoll cushion. The pipette is slowly moved up the tube allowing layering with minimal disturbance of the 40–80% Percoll interface. The 40% Percoll solution is indicated as grey circles. **b** The location of cellular debris, broken chloroplasts, intact chloroplasts, and starch granules in 40–80% Percoll-Ficoll-PEG step gradient after centrifugation is shown. **c** The integrity of freshly isolated chloroplasts in 1X HS buffer was assessed using the Leica SP5 confocal microscope using standard FITC filters at 40× magnification. Scale bar = 10 µm

Use two Percoll gradients for one 60-g chloroplast preparation to avoid over-loading the gradients. Use a wide-bore 1-mL tip and Pipetman (P1000) to gently overlay half of the crude chloroplast suspension onto a Percoll step-gradient (Fig. 2B). The gradient is placed in a test-tube rack immersed in an ice slush bath. Repeat for additional samples. Transport the gradients in the ice bath to the high-speed centrifuge. Wipe the condensation off the tubes, place the tubes in prechilled 30-mL tube adaptors and rubber sleeves within the JS5.3 centrifuge. Centrifuge the gradients at 4200 g for 5 min at 4 °C. The rotor is stopped with the brake. Carefully transfer the gradients to the test-tube rack in the ice bucket.

Place a Percoll gradient in a rack on the cold room benchtop. Using a sterile Pasteur pipette and the in-house aspiration system, gently remove as much of the aqueous upper layer and 40% Percoll layer as possible. The top layer contains cell debris and the aqueous-40% Percoll interface primarily contains broken chloroplasts. Avoid the bright green band at the 40%-80% Percoll interface; this band contains the intact chloroplasts. Using a P1000 and a wide-bore 1-mL tip, transfer the chloroplasts (> 1–2 mL) to a clean 30-mL centrifuge tube. Do not disrupt the starch pellet at the bottom of the tube. Repeat for the remaining gradients. To the six 30-mL tubes (two tubes per 60-g preparation), add 10 to 20 volumes of chilled 1X HS buffer (approximately 20 mL). Seal the tube with parafilm and mix gently by inversion. Remove parafilm and place centrifuge tubes in the JS 5.3 rotor using the 30-mL tube adaptors and rubber sleeves. Spin at 2400*g* for 2 min at 4 °C. Return the tubes to the cold room and carefully pour off the supernatant into a 500-mL beaker and discard.

Gently resuspend the chloroplast pellet in 750 μL of cold 1X HS buffer using a P1000 and wide-bore 1-mL tip and transfer to a sterile 2-mL Axygen microfuge tube. Combine the two pellets from the same 60-g chloroplast preparation in a single 2-mL Axygen microfuge tube. Adjust the volume of chloroplasts to 2 mL with cold 1X HS buffer, mix gently by pipetting up and down. Store chloroplasts at − 20 °C until use. When establishing the protocol, it is important to remove samples (prior to freezing) to verify: (1) chloroplast integrity (50 μL), (2) assess yields by measuring chlorophyll levels (2–5 μL), and (3) determine proteins yields and the quality of chloroplast subfraction by immunoblots (500 μL). Typical chloroplast yields are 26.5 µg Chl/g FW of tomato leaves (Table 1). Over 89% of the chloroplasts are intact using this protocol.

### Stroma extraction protocol

#### General comments

This protocol is based on the 2007 van Wijk et al. protocol [42] with two changes. First, the MgCl_2_ concentration was reduced from 5.0 mM to 2.5 mM; this facilitated better osmotic lysis of tomato chloroplasts. Second, a different proteinase inhibitor cocktail was used. The scheme for extraction of chloroplast stromal proteins is outlined in Fig. 1 and a worksheet for the protocol is provided in Additional file 2: Table S2. Typical yields of stromal protein were 7.95 µg protein/g FW tomato leaves (Table 1; leaf prep #3). A consistent relationship between chlorophyll and protein yields per g FW was seen in all stages of this protocol development with ~ 0.3 µg stromal protein/µg Chl.

#### Preparatory steps

Chill rotors, adaptors and centrifuge tubes (One day prior to stroma extraction)

Prepare the Amicon Ultra 3 K filtration units (1 h prior to chloroplast lysis): Prepare the Amicon Ultra 3 K-filtration unit according to manufacturer’s recommendations. Add 2 mL of sterile deionized water to the Amicon Ultra 3 K filter reservoir. The filter system is centrifuged at 3800 g for 20–25 min at 4 °C in a JS 5.3 rotor using the 30-mL rotor adaptor with sleeves for 15-mL tubes. At this time, approximately 300 µL of water will remain in the chamber; store unit on ice until the stromal extraction is completed. The last steps of Amicon filtration unit preparation are described in the protocol below.

Thaw chloroplasts (1 h prior to chloroplast lysis): Remove chloroplasts from -20 °C freezer and thaw on ice without agitation for 1 h.

Thaw the proteinase inhibitor cocktail (15 min prior to chloroplast lysis): Remove protease inhibitor cocktail from -20 °C freezer and thaw at room temperature.

Prepare the chloroplast lysis buffer (immediately prior to use): Prepare 5 mL of lysis buffer (10 mM HEPES–KOH (pH 8), 2.5 mM MgCl_2_ and 1% (v/v) protease inhibitor cocktail) in sterile deionized water in a 15-mL disposable tube. Use the 1 M HEPES–KOH (pH 8) and 1 M MgCl_2_ stocks. Immediately prior to use, add the thawed protease inhibitor cocktail. Store on ice until use.

### Chloroplast lysis

Pellet thawed chloroplasts by centrifugation at 4 °C for 5 min at 1200 g in a microfuge. Using a P1000, carefully remove and discard the supernatant. Resuspend the chloroplast pellet in 1 mL of chloroplast lysis buffer by vigorous vortexing (~ 1 min) at room temperature. Incubate the chloroplasts for 60 min on ice allowing the chloroplasts to swell and burst. Transfer the suspension to a 2-mL Tenbroeck tissue grinder. Apply three strokes with intermittent rotation of the piston. Transfer the suspension to a 2-mL Axygen microfuge tube. Repeat with additional chloroplast preparations. Rinse the Tenbroeck tissue grinder between different chloroplast preparations.

Centrifuge the suspension at 4 °C for 20 min at 10,000 g in a microfuge to pellet chloroplast membranes. Transfer the supernatant (stroma) to a 3.5-mL polycarbonate ultracentrifuge tube. Freeze the pellet (membranes) at -20 °C for future analyses. Mark the lip of each tube with a marker and orient mark towards the outer rim of the rotor to facilitate locating the minute membrane pellet after ultra-centrifugation. Balance the ultracentrifuge tubes using chloroplast lysis buffer if needed. Centrifuge the supernatant at 300,000 g for 20 min at 4 °C in a TLA-100.3 fixed-angle rotor using a Beckman Coulter Optima™ MAX-TL ultracentrifuge.

Complete preparation of the Amicon Ultra 3 K filtration unit. Transfer the water in the chamber using a P1000 to a waste container (100-mL beaker). Transfer the filtrate in the unit’s reservoir to the waste container. Reassemble filtration unit and immediately load sample as directed below. Do not allow the filters to dry out.

Using a P1000, remove the supernatant (the stroma) avoiding the membrane pellet. Load the stroma (~ 850–900 µL) onto a prepared Amicon Ultra 3 K filter and centrifuge at 4000 g for 50 min at 4 °C in a JS5.3 rotor with 30-mL adaptors and 15-mL rubber sleeves. Peptides and proteins with masses less than 3 kDa are recovered in the effluent and can be discarded or stored at -20 °C for future analyses of peptides. Invert the filtration device (per manufacturer’s instructions) and centrifuge for 2 min at 1000 g at 4 °C to recover the stromal proteins with masses greater than 3 kDa. Approximately 250 µL of stroma is typically recovered. Transfer stroma to a 1.5-mL microfuge tube. Store stromal extracts at -20 °C until use. Prior to freezing, remove a 2-µL sample for protein quantification. When characterizing the system, larger samples were taken for protein isolation and characterization by SDS-PAGE and immunoblots.

### Protein extraction, quantification and immunoblots

Proteins from total leaf extracts, intact chloroplasts, and stromal and non-soluble chloroplast proteins were isolated using the methods described in [61]. Proteins were quantified by a modified Bradford assay (BioRad Laboratories Inc., Hercules, CA) using bovine γ-globulin standard (BioRad) as described in [62]. Proteins were resolved on 12% SDS polyacrylamide gels by electrophoresis and the gel was silver-stained according to [61]. For immunoblots, proteins were transferred to Whatman Protran BA85 nitrocellulose membranes.

For immunoblots with stromal heat shock protein 70 (HSP 70), light-harvesting chlorophyll *a/b* protein (LHCP) and oxygen-evolving complex 23 (OEC 23) antisera, membranes were blocked with 5% milk in TTBS (0.6% Tween-20, 20 mM Tris–HCl (7.5), 500 mM NaCl) for 1 h. Membranes were washed three times with TTBS and incubated for 1 h with primary antisera and subsequently washed three times with TTBS. Antisera were diluted as follows: HSP 70 (1:10,000), OEC 23 (1:10,000), and LHCP (1:20,000). The goat anti-rabbit IgG secondary antiserum with horseradish peroxidase label (Pierce) was diluted 1:50,000 in TTBS. Membranes were incubated with the secondary antiserum for 1 h, and washed three times in TTBS; chloroplast marker-protein blots were then washed once with 1X TBS for 5 min. Membranes were incubated for 5 min with SuperSignal West Pico Chemiluminescent Substrate per vendor recommendations (Thermo Scientific). The HSP 70 antiserum was raised against gel-purified HSP70 isolated from pea chloroplast stroma [63]. The LHCP antiserum was raised against purified pea LHCP II [64]. The OEC23 antiserum was raised against recombinant pea OE23 recovered from inclusion bodies (Kenneth Cline, personal communication) used in [65]. All antisera were provided by Kenneth Cline (U Florida).

For immunoblots with the cytosolic ribosomal protein S6 (RPS6) antiserum [66], membranes were blocked with 5% milk in PBS (1% Tween-20, 100 mM Na_2_HPO_4_.7H_2_O, 14 mM KH_2_PO_4_, 26.8 mM KCl, 1.37 M NaCl) for 1 h. Membranes were washed three times with PBS. Blots were incubated for 1 h with primary antisera (1:5000 dilution) and subsequently washed with three times with PBS. The remaining steps of immunoblot processing was similar to that used for HSP70, OEC23, and LHCP, with the exception that PBS was used instead of TTBS. The RPS6 polyclonal antiserum was prepared against recombinant maize RPS6 produced in *Escherichia coli* and provided by Julia Bailey Serres (UC Riverside).

### Chlorophyll measurements

Chlorophyll was extracted from samples using acetone and concentrations were determined as by Lichtenthaler [67].

### Microscopy

Freshly-isolated chloroplasts were visualized using the Leica SP5 confocal microscope using standard FITC filters at 40× magnification at the UC Riverside Microscopy and Imaging Core. The number of intact and ruptured chloroplasts in a field were counted and the percentage of intact chloroplasts calculated.

### Proteomics analysis

Five stromal protein samples (100 µg) from independent experiments were precipitated in 80% acetone, washed in 100% methanol and dried. Protein pellets were resuspended in 100 µL trypsin solution (10 µg/mL trypsin, 50 mM ammonium bicarbonate (pH 8), 10% acetonitrile) and incubated at 37 °C overnight.

To enhance identification of less abundant stromal proteins, three independent stromal protein preparations (100 µg) were fractionated by 12% SDS-PAGE and stained with Coomassie Blue R-250 [68, 69]. The gel section containing the most abundant proteins (50–70 kDa) was excised and discarded. Three gel sections with proteins of  > 70 kDa, 50 kDa to 20 kDa, and < 20 kDa, respectively, were collected, minced, destained with 50 mM ammonium bicarbonate in 50% acetonitrile, dehydrated with 100% acetonitrile, and air-dried. These dry-gel samples were soaked with sufficient volume of trypsin solution (10 µg/mL trypsin, 50 mM ammonium bicarbonate, pH 8) and incubated overnight at 37 °C. After trypsin digestion, the five acetone-precipitated and three gel-extracted stromal protein samples were analyzed by nanoLC-MS/MS.

A MudPIT approach was employed to analyze the trypsin-treated samples and details are provided in [70]. Briefly, a two-dimension nanoAcquity UPLC (Waters, Milford, MA) and an Orbitrap Fusion MS (Thermo Scientific, San Jose, CA) were configured to perform online 2D-nanoLC-MS/MS analysis. 2D-nanoLC was operated with a 2D-dilution method that is configured with nanoAcquity UPLC. The first dimension LC fractionation used 20 mM ammonium formate (pH 10) and acetonitrile. Five fractions were eluted using 13%, 18%, 21.5%, 27%, and 50% of acetonitrile. The second dimension nanoUPLC method was described previously [70].

Orbitrap Fusion MS method was based on a data-dependent acquisition (DDA) survey using a nanoESI source as described in [68]. Orbitrap mass analyzer was used for the MS1 scan. For the MS2 scan, the Ion-Trap mass analyzer was used in a rapid scan mode. Only precursor ions with intensity 10,000 or higher were selected for MS2 scan. Sequence of individual MS2 scanning was from most-intense to least-intense precursor ions. Higher-energy CID (HCD) was used for fragmentation activation, quadrupole was used for precursor isolation and MS2 mass range was set auto/normal with first mass set at 120 m/z.

The raw MS files were processed and analyzed using Proteome Discoverer version 2.1 (Thermo Scientific, San Jose, CA). Sequest HT search engine was used to match all MS data to the deduced proteome of tomato (ITAG2.4) and our tomato protein Atlas (see below). The search parameters were the following: trypsin with two missed cleavages, minimal peptide length of six amino acids, MS1 mass tolerance 20 ppm, MS2 mass tolerance 0.6 Da, and variable modifications included Gln → pyro-Glu (N-term Q), oxidation (M), and N-terminal acetylation.

Proteins were identified using the deduced tomato proteome [71] based on the criteria detailed in Bhattacharya et al. [68] (Fig. 1). Briefly, all identified proteins (1% FDR) were manually curated. Five independent protein localization algorithms (ChloroP, TargetP, Predotar, WolfPSort, and YLoc) were used to assemble a tomato plastid protein dataset (Atlas) [72–76], which was used to predict subcellular localization of tomato proteins [68]. Based on Arabidopsis homologs, putative localizations of tomato proteins were inferred by the Plant Proteome Database (PPDB; http://ppdb.tc.cornell.edu/) [77], the Plastid Protein Database (plprot; http://www.plprot.ethz.ch/) [78], and Subcellular Localization Database for *A. thaliana* (SUBA4; http://suba.live/) [79]. Data from the primary literature and/or The Arabidopsis Information Resource site (TAIR; https://www.arabidopsis.org/) [80], and Mercator and Mapman BIN ontologies (http://www.plabipd.de/portal/mercator-sequence-annotation/) [81] also aided in protein curation.

Peptide spectral matches (PSMs) and frequency of detection in tomato eight stromal samples were a first criteria for inclusion/exclusion of the tomato chloroplast stromal proteome. Proteins that were detected once with 1 PSM, identified with a single peptide (≥ 2 PSM) or sporadically identified (in less than 40% of the samples analyzed and ≥ 2 PSM) were removed from consideration; the exceptions were proteins that had empirical evidence for residence within the chloroplast. Proteins that reproducibly co-isolated with tomato chloroplast stromal proteins were identified. These proteins were predicted to reside in other organellar compartments by the tomato protein Atlas and/or had Arabidopsis homologs that had empirical data for a non-plastid localization.

Relative protein abundance was calculated based on normalized spectral abundance factors (NSAF) [82, 83]. The spectral abundance factor (SAF) was calculated for each protein. The SAF is the PSMs divided by the number of amino acid residues in a protein. The NSAF is calculated by dividing the SAF for an individual protein by the sum of SAFs for all proteins (1% FDR) and multiplying by 10^3^.

### Comments

#### Overview of the tomato chloroplast isolation method

Numerous methods for isolation of intact plastids and sub-fractionation of chloroplast compartments for proteomics studies in Arabidopsis populate our literature today [42, 46, 84]. In addition, robust methods have been developed for isolation of metabolomics- and proteomics-grade chromoplasts of tomato fruit [47–51, 58] (Additional file 1: Table S1). While protocols for isolating chloroplasts for DNA isolation, enzymatic assays and protein import assays have been described [53–57, 85], rather surprisingly, few chloroplast large-scale proteomics studies have been reported for tomato leaves [52] (Additional file 1: Table S1). Therefore, a high-yield method for intact chloroplast isolation and methods for recovery of the membrane and stromal fractions of chloroplasts for proteomics analyses for tomato was needed.

Our protocol for chloroplast isolation builds upon methods developed for Arabidopsis chloroplasts [42] and incorporates many recommendations from foundational studies in spinach and pea [86, 87]. Our methods for proteomics-grade tomato chloroplasts are more similar to those used by van Wijk et al. [42] for Arabidopsis than the methods previously developed for isolation of chloroplasts from tomato leaves and chromoplasts from tomato fruit, as we used a tissue to buffer ratio of 1:10 and Percoll-Ficoll-PEG step gradients, which had not been previously used for tomato chloroplast isolation (Additional file 1: Table S1). However, unlike the Arabidopsis protocol, our tissue grinding buffer: (1) did not use the anti-oxidant cysteine; (2) included 1 mM MgCl_2_ and 1 mM MnCl_2_ similar to several tomato protocols, and (3) included fivefold higher BSA (0.25%). The general scheme for chloroplast isolation, stroma extraction and proteomics processing and data analysis are provided in Fig. 1 and detailed interactive workflow sheets are found in Additional file 2: Table S2.

Briefly, the deveined tomato leaf homogenate is centrifuged; the pellet contains both intact and broken chloroplasts. This pellet is carefully resuspended and overlaid on the 40%-80% Percoll-Ficoll-PEG step gradient (Fig. 2a). Intact chloroplasts are found at the 40% and 80% Percoll interface (Fig. 2b). Broken chloroplasts and debris are located at the aqueous/40% Percoll interface and starch is located below the 80% Percoll layer. It is clear based on the size of the bands at the two interfaces (Fig. 2b), a majority of chloroplasts recovered from our protocol are intact. Microscopic examination of the 40%-80% interface band shows high-quality intact chloroplasts with over 89% of intact chloroplasts (Fig. 2c).

In the process of refining our chloroplast stroma isolation method, we discovered several parameters that markedly increased tomato chloroplast yield. First, only young, undamaged leaves from 4- to 5-week old tomato plants are used. These leaves are tender and many are expanding and therefore provide best yields. Second, several parts of the homogenization protocol are critical for high-yields and intact chloroplasts. We found that the slurry status of the 1X Grinding Buffer (1 part ice: 1 part liquid) enhanced the yield of intact chloroplasts (Table 1). If the Grinding Buffer is too watery, there is excess chloroplast breakage and stroma is lost; if the buffer is too icy, there is insufficient cell breakage and chloroplasts are not released. Third, the volume of tissue and buffer relative to the blender cup size is critical. If the blender cup is too full, insufficient homogenization occurs; not full enough, foaming (protein denaturation) and excess plastid breakage occurs. Fourth, as well established in the literature [87], the duration of the blender pulse is critical and empirical determination of the optimal blender settings are essential. We found that two 2-sec blender pulses released tomato cell content and retained chloroplast integrity. Another unique feature of our method is that additional leaf tissue was added after the first 2-sec pulse. Unlike the van Wijk et al. method [42], MgCl_2_ and MnCl_2_ are often included in homogenization/grinding buffers, as these ions are important for recovery of biologically active chloroplasts [87] (Additional file 1: Table S1). Finally, we also found that by decreasing MgCl_2_ in the chloroplast lysis buffer from 5 mM [42] to 2.5 mM MgCl_2_ a more complete osmotic rupture of tomato chloroplasts was achieved. With these modifications, typical chloroplast protein yields from tomato leaves yielded 0.3 μg stromal protein per μg of total chlorophyll, with the recovery of ~ 8 µg stromal protein/g FW tomato leaves (Table 1); this is 79-fold higher than the stromal protein yields reported by Hall et al. [46].

#### Integrity of proteins and immunoblot analysis of the stromal chloroplast fractions

**Fig. 3:**
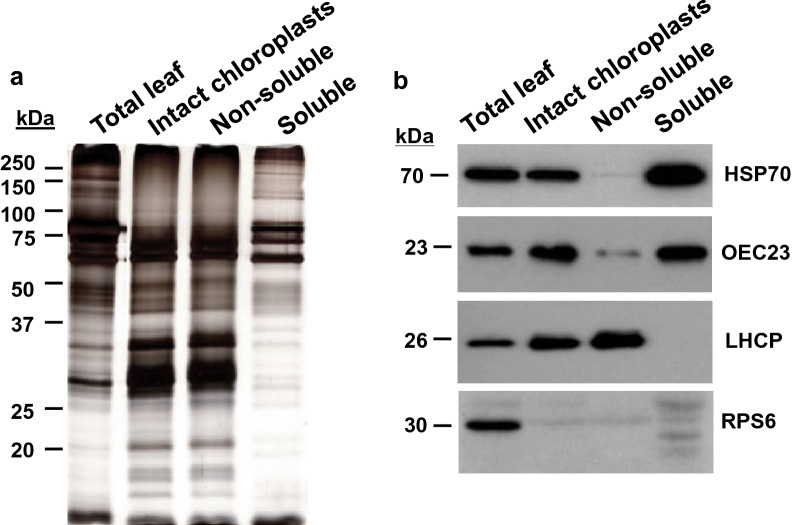
Silver-stained SDS–polyacrylamide gels and immunoblots with protein fractions from the chloroplast stroma isolation protocol. Total leaf proteins (homogenate), intact chloroplasts (from the 40–80% Percoll interface), and non-soluble membranes and stromal proteins released after osmotic lysis of chloroplasts are shown. **a** Equal amounts of protein (1 µg) from each fraction were loaded onto 12% SDS–polyacrylamide gels and silver stained. Masses of molecular weight markers are shown in kDa. **b** Protein blots were incubated with antisera to proteins known to reside in different chloroplast subcellular compartments and the cytosol. Due the differences in abundance of each protein in the different protein fraction and an antisera’s ability to detection tomato proteins, different amounts of protein were loaded per lane: stromal heat-shock protein 70 (HSP70; 12.5 µg), lumenal oxygen-evolving complex (OEC23; 1 µg), thylakoid membrane protein light-harvesting complex (LHCP; 1 µg), and cytosolic ribosomal protein S6 (RPS6; 50 µg). The RPS6 antisera cross-reacts with several tomato proteins. The 30-kDa RPS6 protein is solely found the total leaf homogenate; several of its cross-reacting proteins are enriched during the steps used for chloroplast stromal protein isolation. The mass (kDa) of each protein is shown

To evaluate the integrity of proteins in the leaf homogenate, intact chloroplasts and in the non-soluble and stromal fractions after chloroplast rupture, proteins were fractionated by 12% SDS-PAGE and silver stained (Fig. 3). Proteins ranging from over 150 kDa to under 20 kDa were resolved indicating the high quality of proteins recovered at different stages in the tomato leaf chloroplast protocol. The purified chloroplast extracts were enriched for a subset of the proteins in the total leaf extracts (Fig. 3a). Furthermore, a majority of the abundant proteins found in intact chloroplasts were also present in the non-soluble, membrane fraction after chloroplast lysis. In contrast, the stromal protein fraction is distinct with a small number of superabundant proteins in the 58- to 77-kDa range.

To evaluate the efficacy of our chloroplast and stroma isolation methods, we determined the levels of three proteins that are known to reside in the chloroplast stroma (heat shock protein 70; HSP70), lumen (oxygen evolving complex 23; OEC23), and thylakoid membranes (light harvesting complex proteins; LHCP), as well as one cytosolic protein (ribosomal protein S6; RPS6). In immunoblots, all four proteins were readily detected in total leaf extracts (Fig. 3b). While HSP70, OEC23 and LHCP were detected in isolated chloroplasts, the abundant cytosolic RPS6 was below the level of detection. It should be noted that while this antiserum had high specificity for the 30-kDa RPS6 in maize roots [66], numerous cross-reactive proteins were detected in tomato leaves. However, the 30-kDa RPS6 was the most strongly detected protein and was only identified in leaf homogenates (total leaf protein). These immunoblot data indicate that the chloroplasts are largely free of cytosolic protein contamination. Our proteomics data also supports this result as the cytosolic RPS6 was detected in two of our eight samples with one unique peptide (Additional file 3: Table S3).

Comparison of the non-soluble and stromal protein fractions from isolated chloroplasts showed that the thylakoid-associated LHCP was detected only in the non-soluble fraction, which is enriched for chloroplast membranes including the thylakoid membrane and envelope’s inner and outer membranes (Fig. 3b) [88, 89]. Small amounts of the lumenal OEC23 were also detected in the non-soluble fraction, consistent with OEC23 being an extrinsic protein that associates with OEC33 and OEC16 at the thylakoid membrane for their role in oxygen evolution [90, 91].

Both stromal- and lumen-localized proteins (HSP70 and OEC23, respectively) were detected in the stromal protein samples via immunoblots [90–92]. These data suggested that the stromal extract contained soluble lumenal proteins. The chloroplast lumenal proteome is not complex ranging from 80 to 200 proteins [93–95]; 45 proteins designated as thylakoid peripheral or lumenal proteins in PPDB [77] were detected in the stromal proteome, representing 3.5% of the stromal proteome [68].

#### NanoLC-MS/MS analysis identifies 254 non-plastid co-isolating proteins in the chloroplast stromal proteome

**Fig. 4:**
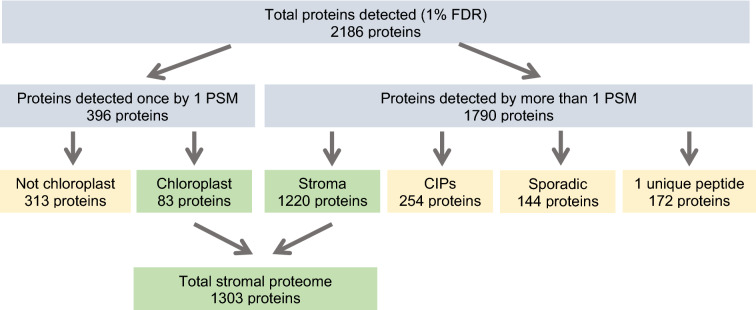
Classification of 1% FDR proteins identified in tomato chloroplast stromal extracts. The 2186 proteins identified in the tomato chloroplast stromal extracts are shown based on their designated categories. The chloroplast stromal proteome has 1303 chloroplast proteins [68]. There were 254 co-isolating proteins (CIPs) that were reproducibly detected. Finally, proteins that were considered contaminants were detected: (1) once with one PSM, (2) with a single peptide (≥ 2 PSMs), or (3) sporadically (≥ 2 PSMs)

We analyzed a total of eight stromal chloroplast protein samples by nanoLC-MS/MS (Fig. 1). In five samples, proteins were acetone precipitated. Three other protein samples were fractionated by SDS-PAGE to remove the superabundant chloroplast proteins in the 50–70 kDa range. Proteins were trypsin digested and analyzed by nanoLC-MS/MS. We identified a total of 2186 proteins (FDR 1%) in tomato’s stromal proteome (Fig. 4) [68]. Manual curation of these proteins was performed using our tomato chloroplast protein Atlas [68], which predicted tomato protein localization using five published algorithms [72–76]. In addition, TAIR and three protein databases (PPDB, plprot and SUBA4) provided theoretical predictions and/or empirical data of the Arabidopsis homolog’s location [77–80].

In the eight stromal samples, 396 proteins were detected once with 1 PSM. Of these proteins, 83 proteins were known to be located within the chloroplast. The remaining 313 proteins had no evidence for chloroplast localization and were classified as low-level contaminants and were removed from further consideration (Fig. 4). Using conservative criteria to identify stromal proteins, we removed 172 proteins identified by one unique peptide (Additional file 3: Table S3; Fig. 4). These proteins had no empirical data to support their localization in the chloroplast based on Arabidopsis homologs (PPDB, plprot and SUBA4 evidence) or the tomato chloroplast protein Atlas. In addition, 144 proteins were identified by more than one peptide but were detected sporadically (one to three times in our eight samples); these proteins were designated as low-level contaminants and not considered further (Additional file 4: Table S4; Fig. 4). In some cases, the PSMs for the sporadically identified proteins were high. For example, a cytosolic HSP70 protein was detected twice in eight samples with a total of 54 PSMs. A summary of the subcellular localization of the proteins identified by one peptide and the sporadically identified proteins is provided in Table 2. Their distribution in the cytosolic, endomembrane, nuclear, mitochondrial, peroxisomal, and plasma membrane compartments was similar.

**Table 2:** Comparison of deduced protein localization for the 254 co-isolating proteins, proteins detected by one unique peptide, and sporadically identified proteins

Location^a^	Co-isolating proteins^b^			“1 unique peptide” proteins^c^			“Sporadically identified” proteins^d^		
	# proteins	%	% total proteins identified	# proteins	%	% total proteins identified	# proteins	%	% total proteins identified
Cytosol	92	36.22	4.21	59	34.10	2.70	52	36.11	2.38
Endomembrane	45	17.72	2.06	25	14.45	1.14	27	18.75	1.24
Mitochondrion	52	20.47	2.38	25	14.45	1.14	21	14.58	0.96
Nucleus	37	14.57	1.69	32	18.50	1.46	30	20.83	1.37
Peroxisome	19	7.48	0.87	5	2.89	0.23	4	2.78	0.18
Plasma membrane	4	1.57	0.18	9	5.20	0.41	1	0.69	0.05
Unknown/Multiple	5	1.97	0.23	17	9.83	0.78	9	6.25	0.41
Total	254	100.00	11.62	172	99.42	7.87	144	100.00	6.59

^a^Based on predictions for the localization of the the tomato proteins by TargetP, ChloroP, Predotar, WoLF PSORT, and YLoc and the known localization (PPDB, plprot, SUBA4) and functions of Arabidopsis homologs proteins (TAIR); the locations of some proteins had multiple subcellular locations or their location could not be inferred

^b^The identity and putative locations of the proteins that reproducibly co-isolated (CIPs) with the tomato chloroplast stroma are found in Additional file 5: Table S5

^c^The identity and putative locations of the proteins identified by a single unique peptide are found in Additional file 3: Table S3

^d^The identity and putative locations of the proteins that were sporadically identified in the chloroplast stroma are found in Additional file 4: Table S4

Of the remaining 1557 identified proteins (FDR 1%), our analyses indicated that there were 1303 high-confidence proteins in the stromal chloroplast proteome [68] and there were 254 reproducibly detected, co-isolating proteins (CIPs), which we discuss here (Additional file 5: Table S5; Fig. 4). These CIPs could: (1) be reflective of the inadvertent co-isolation of small quantities of other organelles; (2) report the extensive and dynamic interactions of chloroplasts with other organelles (e.g., the endoplasmic reticulum, peroxisomes, mitochondria, and nucleus) [31, 96–104] or (3) provide empirical evidence for dual localization of novel proteins in chloroplasts and another organelle.

We assessed the frequency of detection, abundance, and putative localization of the 254 CIPs (Additional file 5: Table S5). All 254 proteins were detected in three or more acetone samples (60–100% reproducibility) and/or two or more PAGE samples (67–100% reproducibility) (Additional file 5: Table S5). For example, 101 of the CIPs (39.8%) were detected in all five acetone samples and 43 of these proteins were detected in all eight samples.

**Fig. 5:**
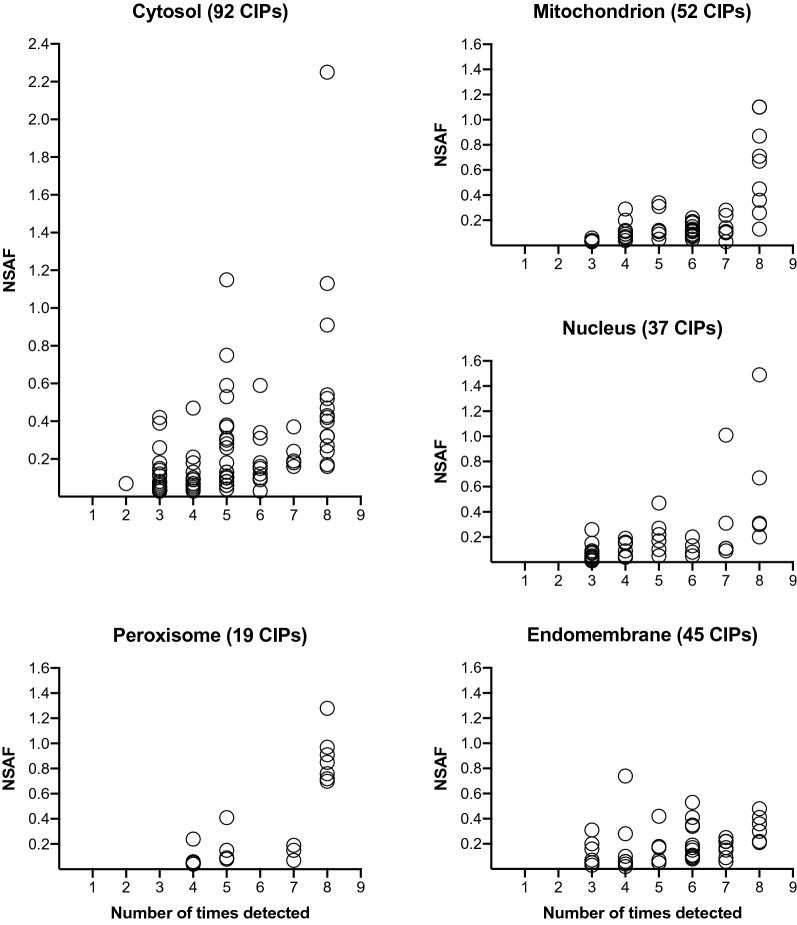
The spectrum of normalized spectral abundance factors (NSAFs) for CIPs with predicted subcellular locations. CIPs predicted to be localized in the cytosol, peroxisome, nucleus, mitochondrion, and endomembrane system are shown. The number of times a protein was detected was plotted against the protein’s NSAF. Each circle represents a single protein. Protein identities, NSAF values and number of times detected can be found in Additional file 4: Table S4

To evaluate the relative abundance of each CIP, we used a protein’s normalized spectral abundance factor (NSAF) [82, 83]. The NSAF was calculated using the SAF (spectral abundance factor) for a protein, which is the number of PSMs divided by the number of amino acid residues for the protein of interest. The protein’s SAF is divided by the sum of all SAFs for all 2186 proteins detected in our studies. The NSAF for the 254 CIPs ranged from 0.01 to 2.25 and CIPs in different organelles had different abundances based on their NSAF ranges (Fig. 5; Additional file 5: Table S5). For perspective, the range of SAFs for the 2186 tomato proteins was from 0.0002 for proteins with 1 PSM to 3.67 for the most abundant protein detected in our eight samples (ribulose-1,5-bisphosphate carboxylase) [68].

The predicted subcellular localization of CIPs was imputed based on the tomato chloroplast Atlas, which provided predictions of the locations of the 254 tomato CIPs (Additional file 5: Table S5). In addition, the PPDB, plprot, and SUBA4 databases provided empirical evidence for Arabidopsis CIP homolog locations. Collectively these data indicated that a majority of the tomato CIP proteins were predicted to reside in the cytosol (36.2%), endomembrane system (17.7%), mitochondrion (20.5%), or nucleus (14.6%) (Fig. 5, 6; Table 2). Three of Arabidopsis homologs (At4g26530, At5g63840, and At5g09590) of the tomato CIPs (Solyc07g065900, Solyc04g049070, and Solyc01g106210, respectively) had empirical evidence for a chloroplast location (Additional file 5: Table S5). However, all three of these Arabidopsis proteins were also detected in other subcellular locations and the tomato Atlas did not predict a chloroplast localization, hence the classification as tomato CIPs. Finally, our analyses also suggested that WoLF PSORT may be a liberal predictor for tomato protein localization in the plastid. WoLF PSORT predicted 62 of the 254 CIPs as chloroplast localized. This prediction was not supported by empirical data for the Arabidopsis CIP homologs, as 31 of these Arabidopsis homologs had a non-chloroplast location (Additional file 5: Table S5).

Embedded within the cytosol, contamination of chloroplasts with abundant cytosolic proteins is anticipated. Accordingly, 92 CIPs with a putative location in the cytosol were identified (Table 2, Additional file 5: Table S5). The most abundant CIP detected in our proteome was the cytosolic phosphoglycerate kinase (PGKc; Solyc07g066600), which was detected in all eight samples and had a total of 235 PSM (Fig. 5; Additional file 5: Table S5). The reason(s) for PGKc co-isolation with tomato’s stromal chloroplast proteins is currently not known. PGKc is important for glycolysis/gluconeogenesis in the cytosol [105]. In pea, PGKc co-localizes with glyceraldehyde-3-P-dehydrogenase, triose-P-isomerase and aldolase providing an opportunity for direct channeling of substrates between the enzymes [106]. However, these additional enzymes were not identified as stromal CIPs suggesting that this complex does not exist in tomato or the protein associations are labile.

Of the remaining cytosolic CIPs, proteins associated with numerous functions were identified. The discovery of the tomato ankyrin-repeat (AKR) protein (Solyc01g104170) as a CIP was not surprising. The Arabidopsis AKR2A homolog (At2g17390) works with a cytosolic HSP17 to target membrane proteins to the plastid outer membrane [107–109]; however, the tomato cytosolic HSP17 homolog was not detected in any of our stromal samples. The most highly represented cytosolic CIPs were those associated with translation, with four elongation factors, two initiation factors, two ribosomal protein subunits, and five tRNA synthetases (Additional file 5: Table S5). When the lists of sporadically identified proteins and proteins identified with one unique peptide were examined, an additional 38 ribosome subunits, five initiation factors and three elongation factors were also identified (Additional file 3: Table S3, Additional file 4: Table S4).

Chloroplasts, peroxisomes, and mitochondria participate in the photorespiratory pathway that catabolizes the products produced by the oxygenation reaction of ribulose-1,5-bisphosphate carboxylase [110]. Electron microscopy and in situ laser analyses have shown that in the light, peroxisomes and mitochondria have intimate and dynamic interactions with chloroplasts and with each other [103, 111]. Chloroplasts may also interact with peroxisomes via dynamic peroxisome membrane extensions called peroxules [112]. Therefore, it is not surprising that 19 peroxisomal proteins and 52 mitochondrial proteins were CIPs (Additional file 5: Table S5, Fig. 5).

Three peroxisome CIPs were photorespiratory enzymes (Additional file 5: Table S5). Hydroxypyruvate reductase (Solyc01g111630), Glutamate:glyoxylate aminotransferase (Solyc05g013380), and Serine:glyoxylate aminotransferase (Solyc12g099930) were identified in all eight samples and were abundant proteins with NSAF scores of 0.7, 0.97 and 0.91, respectively. A byproduct of photorespiration is hydrogen peroxide, which is dissipated by a robust peroxisomal ROS-scavenging system [113]. In accordance, three peroxisomal catalases were CIPs (Additional file 5: Table S5). Two catalases (Solyc02g082760, Solyc12g094620) had high NSAF values (0.72 and 0.78, respectively), while the third catalase (Solyc04g082460) was less abundant (NSAF of 0.24). We also detected monodehydroascorbate reductase (MDHAR, Solyc09g009390) of the glutathione-dependent ROS -scavenging system. MDHAR not abundant (NSAF of 0.08) but it was detected five times. Reumann et al. [83, 113] identified these photorespiratory and ROS-scavenging proteins in purified Arabidopsis peroxisomes; their NSAF values were 5- to 99-fold higher. Although we are extrapolating between two species, the substantial differences in NSAF values for peroxisomes determined by Reumann et al. in Arabidopsis and our stromal proteome in tomato indicates peroxisomal contamination of the tomato chloroplast stromal proteome was minor.

Two peroxisomal enzymes—isocitrate lyase (ICL, Solyc07g052480) and malate dehydrogenase (MD, Solyc02g063490 and Solyc01g106480)—were detected in all eight samples with high NSAF values (Additional file 5: Table S5; Fig. 5). In fact, one MD (Solyc01g106480) had the highest NSAF value (1.28) for all peroxisomal CIPs. ICL and MD have established roles in gluconeogenesis and the glyoxylate cycle in germinating seeds [112] and β-oxidation in young seedlings [114]. In tomato, ICL is detected in fruits and leaves [115] and has been correlated with the peroxisome to glyoxysome transition during leaf senescence [116]. As ICL increases in cotyledons of dark-grown seedlings [115], the high levels of ICL in our samples may reflect the required “dark” incubation of plants prior to chloroplast isolation, which diminishes the number of large starch-filled amyloplasts. It is noteworthy that other enzymes of the glycolytic cycle (e.g. malate synthase, citrate synthase, and aconitase) were not CIPs; although, citrate synthase was detected sporadically (Additional file 4: Table S4).

There were 52 CIPs with a predicted mitochondrial location (Table 2, Additional file 5: Figure S5). NSAFs ranged from 1.1 (malate dehydrogenase and glycine cleavage system T protein) to 0.03 (Additional file 5: Table S5, Fig. 5). Proteins associated with the TCA cycle (14 proteins) and amino acid biosynthesis or catabolism (14 proteins) were enriched in the CIPs.

There is substantial evidence that nuclei and plastids interact [117]. Chloroplasts can be found directly appressed to nuclear envelopes and connected to nuclei via stromules. These direct and yet dynamic communication channels may allow for the exchange of metabolites, H_2_O_2_, and, perhaps, proteins. Thirty-seven proteins with a putative nuclear localization were detected as CIPs (Additional file 5: Table S5; Fig. 5) including seven transcription factors and six proteins associated with RNA biogenesis, which all had low NSAFs ranging from 0.01 to 0.22. The one exception is the abundant glycine-rich RNA-binding protein (Solyc01g109660) with an NSAF of 1.49. Twelve chromatin-associated proteins (i.e., histones, nucleosome assembly and linker proteins, and histone-modifying enzymes) were CIPs with higher NSAFs than most of the transcription factors ranging from 0.03 to 1.01 (for histone H4).

Finally, there is a well-established biochemical continuity between the endoplasmic reticulum and the chloroplast [31]. Therefore, it is not surprising that there were 45 endomembrane system proteins that were identified as CIPs (Table 2, Additional file 5: Table S5, Fig. 5). Overall the ER CIPs were less abundant than CIPs from other organelles, with NSAFs ranging from 0.03 to 0.74 (HSP70) (Fig. 5). Over 80% and 86% of these CIPs had strong support from TargetP (reliability classes 1–3) and Predotar for ER localization, respectively. The majority of the ER CIPs were stress- or defense-related including proteins associated with ER stress-signaling (a calreticulin and UDP-glucose glycosylase), protein folding (HSP70s and a HSP90), peptidases (including N-terminal, C-terminal and internal peptidases), and vacuolar-localized biotic/abiotic defense proteins (trypsin inhibitors, pathogenesis-related proteins, an osmotin, and an AIG-2-like protein).

## Conclusions

Tomato is the most cultivated horticultural crop worldwide, with over 4.7 million hectares planted annually. In US alone, tomatoes are a $2.2 billion industry. Tomato is a model system for studying fruit development [118] and recent insights into the dynamics of the tomato’s plastid proteome during the differentiation of chloroplasts to chromoplasts have provided important insights in these processes [47, 48, 51]. In addition, tomato is a model system for the study of the induction of plant defenses associated with wounding, herbivory and pathogen attack [119].

As chloroplasts are key regulators of stress perception and signal transduction [5, 33] and the site of production of secondary metabolites and plant hormones involved in defense, an understanding of the dynamics of the chloroplast leaf proteome is needed. The protocol provided here provides a detailed method to assure high quality and high yields of intact chloroplasts from tomato leaves suitable for proteomics analysis. As a number of yield-limiting steps were identified in this protocol, the methods can be adapted to virtually any plant species. In conjunction with the tomato nuclear and plastid genome sequences [56, 71], evaluation of changes to the tomato chloroplast proteome, and its sub-organellar fractions, in response to cues during development, as well as abiotic and biotic stress are now possible. Future confirmation of CIP localization using fluorescent reporter fusion proteins will determine if these proteins are imported and localized in more than one organelle or if their co-isolation with chloroplasts solely reflects the known tight apposition of ER, peroxisomes, mitochondria, and nuclei with chloroplasts [103, 111, 112, 117].

## Supplementary information

**Additional file 1: Table S1.** Protocol comparison.

**Additional file 2: Table S2.** Chloroplast Isolation Interactive Worksheet.

**Additional file 3: Table S3.** Proteins detected based on one unique peptide.

**Additional file 4: Table S4.** Proteins that were sporadically identified.

**Additional file 5: Table S5.** Co-isolating proteins (CIPs).

## Data Availability

Not applicable

## Abbreviations

CIP: co-isolating protein
LAP-A: leucine aminopeptidase A
NSAF: normalized spectral abundance factor
PSMs: peptide spectral matches
SAF: spectral abundance factor
